# Detection of Focal Lesions in Epilepsy with [^18^F]UCB-H Synaptic Vesicle Protein 2A PET Imaging

**DOI:** 10.2967/jnumed.125.271505

**Published:** 2026-06

**Authors:** Vanessa Pakula, Julia S. Dorneich, Lukas Frontzkowski, Laura M. Bartos, Nicholas Fearns, Sebastian N. Roemer-Cassiano, Simon Lindner, Andreas Zwergal, Elisabeth Kaufmann, Günter Höglinger, Nicolai Franzmeier, Rudolf A. Werner, Christian Vollmar, Jan Rémi, Matthias Brendel, Johannes Gnörich

**Affiliations:** 1 Department of Nuclear Medicine, LMU University Hospital, Munich, Germany;; 2 Institute for Stroke and Dementia Research, LMU Hospital, LMU Munich, Munich, Germany;; 3 Institute of Clinical Neuroimmunology, LMU University Hospital, LMU Munich, Munich, Germany;; 4 Department of Neurology, LMU University Hospital, LMU Munich, Munich, Germany;; 5 Max Planck School of Cognition, Leipzig, Germany;; 6 German Center for Vertigo and Balance Disorders, LMU University Hospital, LMU Munich, Munich, Germany;; 7 German Center for Neurodegenerative Diseases Munich, Munich, Germany;; 8 Munich Cluster for Systems Neurology (SyNergy), Munich, Germany;; 9 Department of Psychiatry and Neurochemistry, The Sahlgrenska Academy, Institute of Neuroscience and Physiology, University of Gothenburg, Gothenburg, Sweden; and; 10 Russell H. Morgan Department of Radiology and Radiological Sciences, Johns Hopkins School of Medicine, Baltimore, Maryland

**Keywords:** SV2A PET, focal epilepsy, synaptic density, [^18^F]FDG PET

## Abstract

Synaptic vesicle protein 2A (SV2A) is a universal marker of synaptic density. Recent advances in SV2A-targeted radiotracers have opened new windows into synaptic imaging. In focal epilepsy, synaptic dysfunction is a central pathologic feature. Unlike [^18^F]FDG PET, which reflects neuronal metabolism only indirectly, SV2A PET allows for direct quantification of synaptic density. We therefore evaluated [^18^F]UCB-H SV2A PET in comparison to [^18^F]FDG PET in patients with pharmacoresistant, unilateral focal epilepsy, aiming to assess its complementary value to established metabolic imaging. **Methods:** In total, 29 patients with unilateral focal epilepsy underwent both dynamic [^18^F]UCB-H PET (0–60 min) and static [^18^F]FDG PET (30–50 min) imaging. Eight patients were treated with the SV2A-binding medications levetiracetam or brivaracetam. [^18^F]UCB-H PET time–activity curves were extracted from 35 frames across cortical and subcortical regions, and Pearson correlation coefficients with [^18^F]FDG uptake were calculated for each frame to identify the most suitable imaging windows. Voxelwise percentage differences between epileptogenic and contralateral healthy hemispheres were computed to determine lesion severity and volume. Finally, we evaluated gaussian smoothing kernels for minimizing background noise while preserving contrast during lesion detection. **Results:** Treatment with SV2A-binding medications reduced late-phase [^18^F]UCB-H binding up to 75% compared with untreated individuals, demonstrating high target specificity. Framewise correlation analysis in unaffected contralateral hemispheres revealed significant associations between [^18^F]UCB-H and [^18^F]FDG uptake within the 0–10-min and 30–60-min postinjection intervals. These time windows were therefore selected for early- and late-phase analyses, respectively. Within epileptogenic foci, SV2A PET lesion severity correlated with [^18^F]FDG uptake for both early-phase (*r* = 0.61, *P* = 0.0042) and late-phase (*r* = 0.63, *P* = 0.0027) imaging. However, only early-phase SV2A PET lesion volume correlated with [^18^F]FDG lesion volume (*r* = 0.70, *P* = 0.0004), whereas late-phase SV2A PET volume did not. In line, [^18^F]FDG and early-phase [^18^F]UCB-H PET visually showed broad hypometabolic and hypoperfused areas around the epileptogenic zone, whereas late-phase [^18^F]UCB-H PET yielded sharper, high-contrast delineation of synaptic abnormalities. **Conclusion:** Dual-phase [^18^F]UCB-H PET provides complementary perfusion-like and synaptic information in focal epilepsy and shows spatial correspondence with [^18^F]FDG PET while offering more spatially confined synaptic signal changes.

## Visual Abstract

**Figure:**
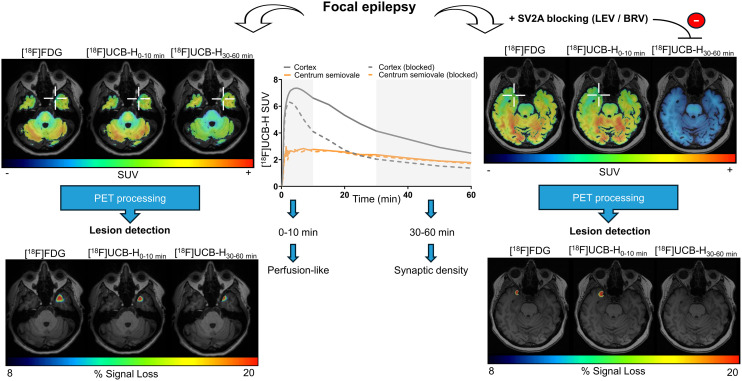

Epilepsy, a chronic neurologic disorder, is characterized by recurrent, unprovoked seizures, with nearly 30% of patients remaining refractory to available therapies (1,2). In focal epilepsy, where seizures originate from a localized brain region, precise delineation of the epileptogenic zone is essential for guiding effective surgical intervention and improving long‐term outcomes (3).

Traditionally, interictal [^18^F]FDG PET has been a cornerstone in presurgical evaluations, especially in patients with inconclusive structural MRI findings (4–6). [^18^F]FDG PET detects areas of hypometabolism that are often associated with dysfunctional, chronically epileptogenic tissue (7). However, [^18^F]FDG measures are inherently indirect and can be confounded by factors such as ictal timing, inflammation, fluctuating blood glucose levels, and other physiologic variations affecting cerebral glucose metabolism (8). In addition to metabolic imaging, interictal perfusion techniques such as [^99m^Tc]HMPAO SPECT have been used to evaluate cerebral blood flow abnormalities in epilepsy (9,10). Although these methods aim to detect hypoperfused regions associated with seizure onset zones, SPECT is limited by lower spatial resolution and more limited quantification compared with PET.

Recent advances in molecular imaging have shifted attention toward more direct targets of synaptic integrity. Synaptic vesicle glycoprotein 2A (SV2A) has emerged as a promising candidate in this context (11). SV2A is an integral presynaptic membrane protein that resides in virtually all synaptic vesicles and plays a critical role in neurotransmission and vesicle trafficking (12,13). Moreover, the clinical relevance of SV2A is underscored by the fact that the antiepileptic drugs levetiracetam and brivaracetam bind to SV2A, thereby linking alterations in SV2A expression to the pathophysiology of epilepsy (14). In contrast to [^18^F]FDG PET, which indirectly reflects neuronal function via metabolism, SV2A PET imaging offers a direct measure of synaptic density, providing more specific insights into the synaptic loss and reorganization that underlie focal epileptogenesis (15).

Several radiotracers have been developed to target SV2A, including [^11^C]UCB-J, [^11^C]UCB-A, and, more recently, [^18^F]UCB-H (16–18). Among these, [^11^C]UCB-J has demonstrated excellent binding specificity and sensitivity across a range of neurodegenerative diseases and epilepsy and is currently the most widely used SV2A tracer (19–21). However, its application is limited by the short half‐life of ^11^C (∼20 min), which restricts its use to facilities equipped with an on-site cyclotron. In contrast, emerging ^18^F-labeled tracers, including [^18^F]SynVesT-2, [^18^F]UCB-J, and [^18^F]UCB-H, offer logistical advantages because of their longer half-life (∼110 min) (22,23). Although these fluorinated tracers are currently used predominantly in research and early-phase clinical investigations, their favorable physical properties make them promising candidates for wider clinical application.

Preclinical studies have shown that [^18^F]UCB-H provides a robust measure of synaptic density in animal models of epilepsy, with its uptake correlating closely with SV2A expression as confirmed by immunohistochemical analyses (24,25). Although SV2A PET imaging has shown promise in identifying epileptogenic regions, its application in human epilepsy remains limited. For instance, a recent study demonstrated reduced [^11^C]UCB-J binding in the seizure onset zones of 12 patients with temporal lobe epilepsy, suggesting that SV2A PET imaging can detect areas of synaptic loss associated with epileptogenic regions (21). Despite these encouraging findings, comprehensive studies directly comparing novel SV2A PET tracers, such as [^18^F]UCB-H, with established imaging modalities, such as [^18^F]FDG PET, in humans remain scarce.

In addition to assessing synaptic density, dynamic SV2A PET imaging offers the potential to capture perfusion-like information during the early postinjection phase. This capability is particularly relevant for interictal perfusion imaging. Dynamic SV2A PET imaging, therefore, presents an opportunity to simultaneously assess both synaptic density and perfusion-like characteristics.

In the present study, we leveraged this dual capability by analyzing both early-phase (perfusion-like) and late-phase (synaptic density) data from dynamic [^18^F]UCB-H PET imaging in a cohort of 29 patients with focal epilepsy. By systematically comparing these findings with [^18^F]FDG PET results, we aimed to evaluate the added diagnostic value of SV2A PET imaging in the presurgical evaluation of epilepsy. To this end, we investigated the methodologic performance of [^18^F]UCB-H by identifying optimal postinjection time windows and image-processing strategies to maximize lesion contrast and reduce background noise, refining its clinical applicability.

## MATERIALS AND METHODS

### Cohort and Study Design

Individuals with drug-resistant, unilateral focal epilepsy (*n* = 29; 16 women, 13 men) with a mean age of 33.6 ± 14.6 y at the time of SV2A PET imaging were recruited from the Department of Neurology, Ludwig-Maximilians University Hospital. The overall lesion site was determined during clinical work-up by an interdisciplinary epilepsy board in accordance with current guidelines, including neuropsychological testing, structural MRI, and electroencephalogram with video monitoring. On the basis of this integrated assessment, overall localization was classified as temporal (*n* = 22), frontal (*n* = 4), temporo-occipital (*n* = 1), frontotemporal (*n* = 1), or temporoparietal (*n* = 1). Lateralization was left-sided in 18 patients and right-sided in 11 patients. Disease duration was available for most patients and averaged 20.1 ± 15.3 y, reflecting a chronic epilepsy population. Of the 29 patients, 8 were receiving or had recently received SV2A-binding antiseizure medication. These patients are referred to throughout the article as the blocked group, defined operationally as current or recent treatment with levetiracetam (1500–4000 mg/d; *n* = 5) or brivaracetam (200 mg/d; *n* = 3). This classification reflects an observational grouping based on medication exposure and does not constitute a controlled pharmacologic blockade or definite quantitative occupancy study. The time interval between [^18^F]FDG and [^18^F]UCB-H scans was 3.3 ± 5.1 mo. Static [^18^F]FDG PET scans were acquired 30–50 min postinjection, with a mean injected activity of 146.7 ± 10.5 MBq. For SV2A PET, patients received dynamic PET imaging over 60 min after the intravenous bolus injection of [^18^F]UCB-H, with a mean injected activity of 185.1 ± 13.6 MBq. The molar activity of [^18^F]UCB-H at the end of the synthesis was 537 ± 270 GBq/µmol. Five patients had previously undergone epilepsy surgery before PET imaging but continued to experience seizures. All participants provided written informed consent before the PET scan. The study was approved by the local ethics committee of the medical faculty of the Ludwig-Maximilians University Munich (application numbers 22-0997 and 163-16) and the German Radiation Protection Authority (application number BfS-ZD3-2024-036-G). A detailed overview of the study population is provided in Supplemental Table 1 (supplemental materials are available at http://jnm.snmjournals.org).

### PET Imaging

Detailed information on PET data acquisition, reconstruction, preprocessing, and analysis is provided in the Supplemental Methods (26–32).

### Statistical Analysis

All statistical analyses were conducted using SPSS (version 29.0; IBM Corp.) and GraphPad Prism (version 10.1.2). Multiple unpaired *t* tests were performed to compare [^18^F]UCB-H PET signals between blocked and unblocked patients across regions of interest, with *P* values corrected for multiple comparisons using the Holm–Šidák method. Pearson correlation coefficients (*r*) were calculated to assess associations between [^18^F]UCB-H PET signals and [^18^F]FDG PET uptake across dynamic frames and brain regions. Correlations were considered significant at a threshold *P* value of less than 0.05. Framewise comparisons between [^18^F]UCB-H and [^18^F]FDG PET were performed separately for SUV and SUV ratios (SUVR) using cerebellar scaling, as appropriate for early and late phases. To derive lesion severity and volume metrics, analyses were restricted to the most affected 90% of voxels within the suprathreshold lesion mask. This approach was chosen to reduce the influence of edge-related noise, partial-volume effects, and residual misregistration at lesion boundaries, which are particularly pronounced in asymmetry-based subtraction maps. Lesion metrics were then compared across tracers using Pearson correlation analyses. Furthermore, lesion volumes and severity differences were assessed using a 1-way ANOVA followed by the Tukey multiple comparison test.

## RESULTS

### Tracer Kinetics of [^18^F]UCB-H SV2A PET in Nonepileptogenic Brain Regions

Before evaluating the diagnostic utility of SV2A PET in focal epilepsy, we first examined the tracer kinetics of [^18^F]UCB-H over the entire scan duration (0–60 min) in the unaffected contralateral hemisphere, both in patients receiving SV2A-binding medication (levetiracetam/brivaracetam) and in unblocked patients. This step served to characterize general tracer behavior and its relationship to glucose metabolism, independent of epileptogenic alterations.

**FIGURE 1.**
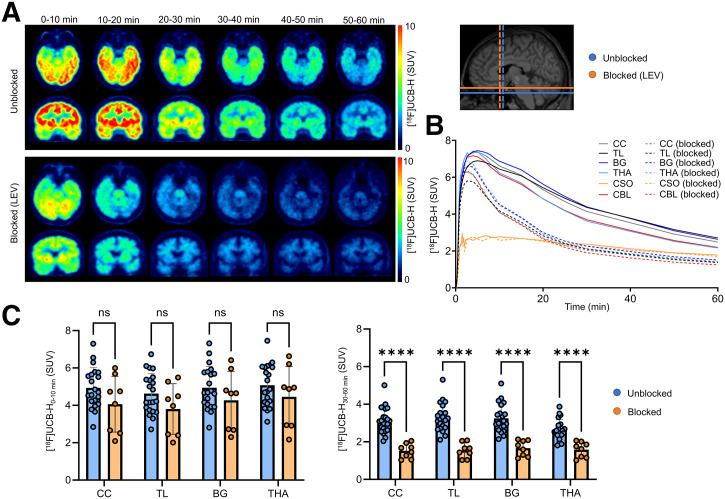
Pharmacokinetics of [^18^F]UCB-H in unaffected contralateral hemisphere. (A) Axial and coronal SUV maps of dynamic [^18^F]UCB-H PET (0–60 min) of 2 representative patients, 1 unblocked and 1 receiving 2000 mg of levetiracetam (LEV), illustrating regional tracer uptake over time. (B) Averaged time–activity curves of [^18^F]UCB-H SUV in unblocked (*n* = 21) and blocked (*n* = 8) patients across representative brain regions. (C) Bar graphs depict group differences between unblocked (blue) and blocked (orange) patients for early-phase (0–10 min, left) and late-phase (30–60 min, right) in 4 representative brain regions: cerebral cortex (CC), temporal lobe (TL), basal ganglia (BG), and thalamus (THA). CBL = cerebellum; CSO = centrum semiovale; ns = not significant. ****P < 0.0001.

We compared averaged [^18^F]UCB-H SUV time–activity curves between both cohorts across multiple regions of interest (Figs. 1A and 1B). In the blocked cohort, [^18^F]UCB-H showed a significant earlier peak (*P* < 0.0001), whereas the peak magnitude was slightly but not significantly lower compared with unblocked subjects (*P* = 0.19). For instance, in the contralateral cerebral cortex, peak activity occurred at 2.7 ± 0.8 min postinjection in blocked patients with an SUV of 6.4 ± 2.3 versus 4.0 ± 0.7 min in unblocked individuals with an SUV of 7.4 ± 1.6 (Fig. 1B). Across all the analyzed regions, a rapid initial tracer influx was followed by a continuous decline in tracer binding. This decline was more pronounced in the blocked cohort, indicating faster tracer washout from brain tissue (Fig. 1B).

Importantly, we observed pronounced reductions of [^18^F]UCB-H PET signals in patients receiving SV2A-targeting medication. Significant reductions in SV2A PET binding during the late imaging window were observed across all examined brain regions, including the cerebral cortex (−51.1%, *P* < 0.0001), temporal lobe (−53.1%, *P* < 0.0001), basal ganglia (−48.8%, *P* < 0.0001), and thalamus (−40.1%, *P* = 0.0002), with peak signal blocking of up to 75% in cortical subregions (*P* < 0.0001). In contrast, no significant differences were detectable during the early imaging phase (Fig. 1C). The centrum semiovale, characterized by the lowest peak uptake and a relatively flat washout profile, showed no significant difference in tracer kinetics between the blocked and unblocked cohort, supporting its suitability as a reference region for SV2A PET quantification (Fig. 1B).

### Regional Relationship Between SV2A PET and Glucose Metabolism

After characterizing early-phase perfusion-like peaks and tracer kinetics in unaffected brain regions, we investigated which [^18^F]UCB-H time frames best reflect glucose metabolism as measured by [^18^F]FDG PET in the same regions. Thus, we compared dynamic single-frame [^18^F]UCB-H PET (*n* = 35; 12 × 5 s, 6 × 10 s, 3 × 20 s, 7 × 60 s, 4 × 300 s, 3 × 600 s) with [^18^F]FDG PET scans acquired during the late static phase (30–50 min).

**FIGURE 2.**
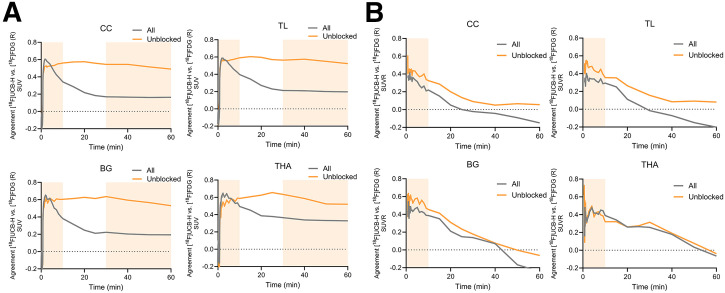
Imaging time-dependent agreement between dynamic [^18^F]UCB-H and [^18^F]FDG PET uptake. (A) Pearson correlation coefficients (*r*) between SUV from individual [^18^F]UCB-H PET frames (0–60 min) and static [^18^F]FDG PET uptake (30–50 min) across 4 brain regions: cerebral cortex (CC), temporal lobe (TL), basal ganglia (BG), and thalamus (THA). (B) Corresponding correlations using SUVR with cerebellum normalization. Curves are shown separately for entire cohort (*n* = 29, gray) and unblocked subgroup (*n* = 21, orange). Shaded orange areas indicate predefined early (0–10 min) and late (30–60 min) time windows used for subsequent analyses.

Significant correlations between [^18^F]UCB-H SUV and [^18^F]FDG SUV uptake in the contralateral cerebral cortex of all 29 patients were observed between frames 11 and 27, corresponding to approximately 1–10 min postinjection. In the subgroup of 21 unblocked patients, correlations became significant shortly after the first minute and quickly reached a stable plateau that persisted throughout the dynamic acquisition (Fig. 2A). When both modalities were scaled to the mean intensity of the cerebellum, significant associations emerged during the initial minutes of the dynamic acquisition, followed by a steady decline over time (Fig. 2B). Combined with the observed average perfusion-like peak, these findings indicate that early-phase SV2A PET imaging should be conducted during the first 10 min postinjection to provide an optimal balance between count statistics and correlation with glucose uptake. Conversely, tracer binding during the late phase (30–60 min) was characterized by stable agreement for SUV across regions, supporting its application for synaptic density assessment. Detailed results, including single-frame correlation coefficients across all brain regions, are provided in Supplemental Tables 2–5.

In the subgroup of 21 patients without pharmacologic SV2A blockading, region-based correlation analyses based on the preselected early-phase (0–10 min) and late-phase (30–60 min) time windows demonstrated strong concordance between [^18^F]UCB-H and [^18^F]FDG PET signals across cortical and subcortical brain regions, using both SUV and SUVR scaling (Supplemental Results; Supplemental Fig. 1). Additionally, intraindividual correlations across 42 Hammers atlas regions in the unaffected hemisphere revealed that 0–10 min [^18^F]UCB-H PET showed the strongest agreement with [^18^F]FDG PET, even under SV2A-blocking conditions, whereas late-phase correlations markedly declined in blocked patients (Supplemental Results; Supplemental Fig. 2). Importantly, the 0–10 min [^18^F]UCB-H SUVR correlations with [^18^F]FDG PET remained robust across alternative normalization strategies, including whole-brain global mean and pons reference regions, and showed strong interreference consistency (Supplemental Results; Supplemental Figs. 3 and 4).

To further support the use of static late-phase [^18^F]UCB-H measures, we next evaluated the agreement between static SUV/SUVR metrics and kinetic outcome parameters. Both total volume of distribution ratio (image-derived input functions) and distribution volume ratio (reference tissue modeling) showed strong regional correlations with static [^18^F]UCB-H SUVR, particularly during the late imaging window (Supplemental Figs. 5 and 6).

### SV2A PET Reveals Unilateral Synaptic Loss in Focal Epilepsy

**FIGURE 3.**
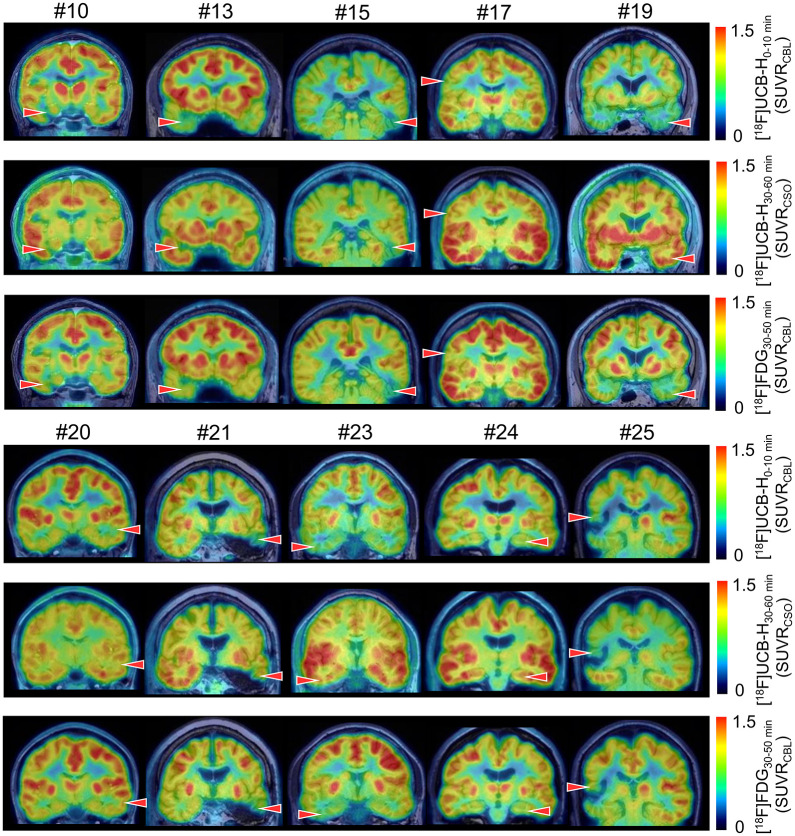
Comparison of early- and late-phase [^18^F]UCB-H images compared with [^18^F]FDG PET. Coronal slices showing early- and late-phase [^18^F]UCB-H PET images alongside [^18^F]FDG PET in 10 representative unblocked patients, overlaid on corresponding T1-weighted MRI. SUVRs were calculated using cerebellar (CBL) scaling for early-phase SV2A PET and [^18^F]FDG PET and centrum semiovale (CSO) scaling for late-phase SV2A PET. Red arrowheads indicate location of epileptogenic lesion identified in each patient.

We next investigated the ability of [^18^F]UCB-H PET to detect synaptic loss within epileptogenic lesions in unblocked patients. From the cohort of 21 patients with unblocked SV2A PET signals, we selected 10 representative individuals with clinically confirmed and visually distinct unilateral reductions in synaptic density for quantitative signal-to-noise analysis, including 1 patient (patient 21) with prior epilepsy surgery and documented persistent seizures despite resection (Fig. 3).

**FIGURE 4.**
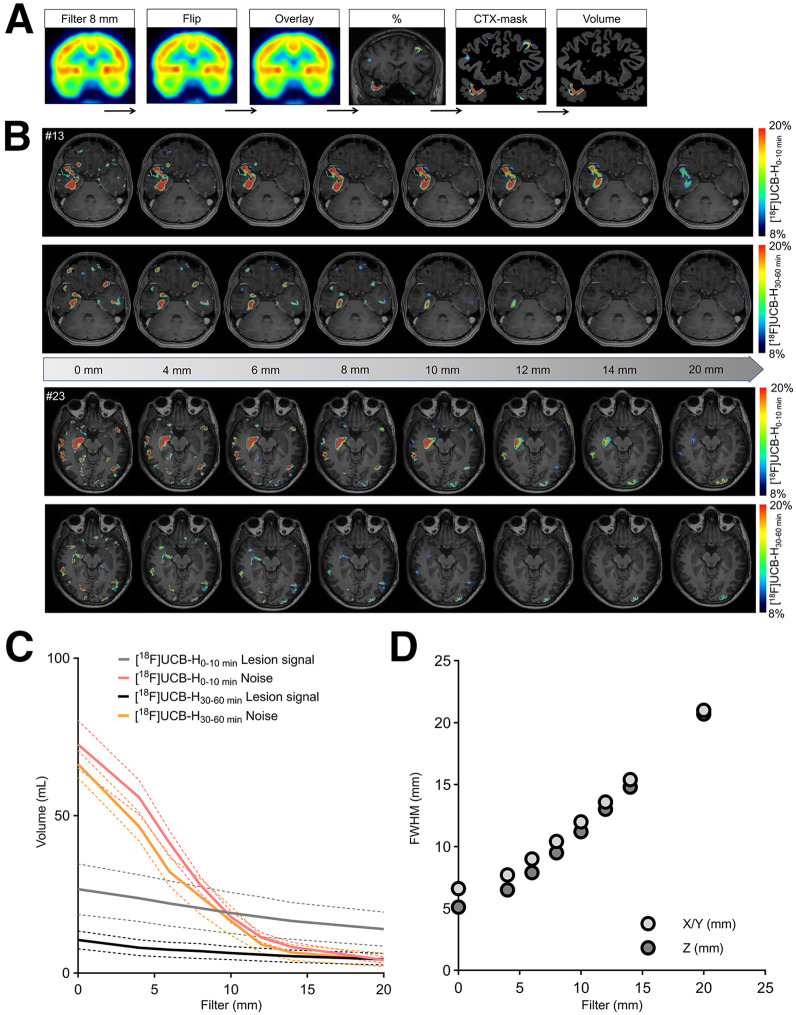
Optimization of postprocessing for lesion detection via asymmetry in SV2A PET. (A) Workflow for identifying epileptogenic foci using mirrored subtraction: raw images were smoothed, flipped across midline, overlaid, subtracted from original, masked to cortical voxels, and quantified volumetrically. (B) Axial slices show voxelwise percent differences between hemispheres across various gaussian smoothing levels (0–20 mm FWHM) in 2 representative patients. (C) Quantification of lesion signal and background noise across filters for early-phase and late-phase [^18^F]UCB-H PET. (D) Relationship between applied gaussian smoothing kernel and resulting PET resolution (FWHM) in *X*/*Y* and *Z* directions.

To enhance lesion contrast and diagnostic performance, we evaluated postprocessing strategies before hemispheric flipping to highlight asymmetric synaptic reductions using the contralateral hemisphere as the internal reference (Fig. 4A). Incremental gaussian smoothing revealed that applying an 8-mm full-width-at-half-maximum (FWHM) filter substantially reduced background noise by approximately 50%, while preserving lesion extent with only minor volume reduction (Figs. 4B and 4C). Based on the intrinsic scanner resolution (6.6 × 6.6 × 5.1 mm), the resulting FWHM increased predictably with the applied gaussian kernel. For instance, an 8-mm filter yielded effective resolutions of 10.4 × 10.4 × 9.5 mm (Fig. 4D). After applying the optimized 8-mm gaussian filter and performing hemispheric flipping, we computed voxelwise percent difference maps between original and mirrored PET images to quantify asymmetry (Fig. 4B).

**FIGURE 5.**
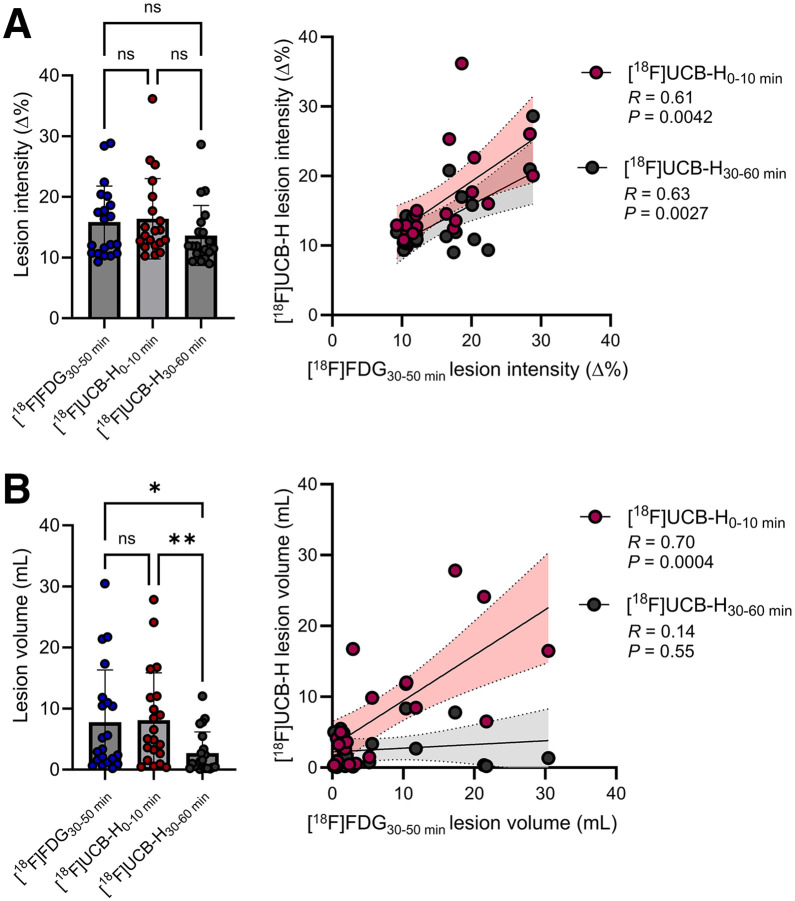
Lesion severity and volume comparison between [^18^F]FDG and [^18^F]UCB-H PET. (A) Lesion severity (Δ%) derived from [^18^F]FDG PET and early- and late-phase [^18^F]UCB-H PET (bar plots) with corresponding cross-modality correlations (scatter plots). (B) Lesion volumes for each modality (bar plots) and their correlations between [^18^F]FDG PET and early- and late-phase [^18^F]UCB-H PET (scatter plots). Error bars indicate SD; shaded areas denote 95% CI. ns = not significant. *P < 0.05, **P < 0.01.

Using these insights, we next compared early- and late-phase [^18^F]UCB-H PET with [^18^F]FDG PET across all unblocked patients to evaluate which phase better reflects the metabolic lesion profile in terms of both signal severity and lesion volume. Focusing on the most affected 90% of lesion voxels, defined by an ipsilateral versus contralateral asymmetry threshold of 8%, lesion severity was comparable across [^18^F]FDG as well as early-phase and late-phase [^18^F]UCB-H, with no significant differences between groups (Fig. 5A). In contrast, lesion volume differed significantly between modalities, with 0–10 min [^18^F]UCB-H (*P* = 0.0072) and [^18^F]FDG (*P* = 0.036) yielding larger volumes than 30–60 min [^18^F]UCB-H. No significant difference was observed between [^18^F]FDG and 0–10 min [^18^F]UCB-H (*P* = 0.97). We then assessed spatial correspondence by correlating lesion intensities between modalities. Significant positive associations were found between [^18^F]FDG and 0–10 min [^18^F]UCB-H (*r* = 0.61, *P* = 0.0042) as well as between [^18^F]FDG and 30–60 min [^18^F]UCB-H (*r* = 0.63, *P* = 0.0027) (Fig. 5A). However, regarding lesion volume, only 0–10 min [^18^F]UCB-H showed a strong and significant correlation with [^18^F]FDG (*r* = 0.70, *P* = 0.0004), whereas no correlation was observed for 30–60 min [^18^F]UCB-H (*r* = 0.14, *P* = 0.55) (Fig. 5B). Importantly, the relative differences between modalities remained consistent across a range of asymmetry thresholds (0%, 5%, 10%) (Supplemental Fig. 7), with the 8% threshold providing a pragmatic trade-off by limiting the contribution of spatially diffuse, low-amplitude asymmetries while preserving sensitivity to the lesion core.

## DISCUSSION

In this study, we provide the first systematic comparison of [^18^F]UCB-H SV2A PET and [^18^F]FDG PET in a clinically well-characterized cohort of patients with unilateral focal epilepsy. By combining dynamic SV2A PET imaging with optimized image-processing strategies, we demonstrate that [^18^F]UCB-H captures both perfusion-like and synaptic density–related aspects of epileptogenic brain changes, complementing metabolic insights provided by [^18^F]FDG PET.

As a major achievement, this study establishes a methodologic framework for disentangling the dual biologic signals embedded in [^18^F]UCB-H PET imaging, early-phase perfusion-like and late-phase synaptic density, in patients with unilateral focal epilepsy. To ensure reliable interpretation of these signals, we first focused on the contralateral, non-epileptogenic hemisphere. This region served as a stable reference to characterize tracer kinetics independent of pathology, enabling the identification of distinct uptake phases and the definition of optimal time windows. This preparatory analysis laid the methodologic groundwork for subsequent lesion-focused evaluations. The 0–10 min [^18^F]UCB-H showed strong spatial concordance with [^18^F]FDG PET in terms of lesion severity and volume. This rationale aligns with the wider early-phase PET literature, particularly in neurodegenerative diseases, where early-frame tau and amyloid PET imaging have been demonstrated to act as a perfusion-like surrogate (33). In contrast, 30–60 min [^18^F]UCB-H captured synaptic density with higher contrast and more precise delineation of the epileptogenic zone, complementing the broader metabolic patterns observed with [^18^F]FDG.

Notably, whereas lesion localization overlapped between 30–60 min [^18^F]UCB-H and [^18^F]FDG PET, [^18^F]FDG consistently revealed larger lesion volumes. This aligns with the view that metabolic alterations involving astrocytic and axonal components (34) can precede detectable synaptic loss (35). Moreover, interictal [^18^F]FDG PET is known to capture not only the seizure onset zone but also the broader irritative zone and downstream networks involved in epileptogenesis (36,37). This may explain the observation of hypometabolic regions extending beyond the presumed seizure onset zone, which has been reported in both temporal and extratemporal lobe epilepsy and has been shown to carry prognostic value for surgical outcome (38). In this context, the more spatially confined abnormalities observed with late-phase SV2A PET could reflect synaptic alterations within epileptogenic tissue rather than network-level metabolic dysfunction and should be interpreted as complementary information rather than a direct surrogate for seizure onset zone definition, pending prospective validation against histopathology and pre- and postoperative imaging.

The dual-phase design also offers a practical benefit in patients undergoing treatment with SV2A-targeting medication. Although a late-phase SV2A signal is markedly reduced under pharmacologic blockade, 0–10 min [^18^F]UCB-H uptake remains largely unaffected, allowing reliable extraction of perfusion-like information even in these cases. Nevertheless, the value of early-phase imaging under a pharmacologic SV2A blockade warrants further validation in larger, dedicated patient cohorts.

Our dynamic acquisition protocol over 60 min enabled a detailed kinetic analysis of [^18^F]UCB-H and, importantly, allowed direct comparison between static and dynamic outcome measures in the present human cohort. We observed strong agreement between late-phase static [^18^F]UCB-H measures and kinetic parameters derived from dynamic modeling, including total volume of distribution ratio and distribution volume ratio, indicating that 30–60 min [^18^F]UCB-H closely approximates dynamic kinetic parameters. This extends prior preclinical observations and provides direct human evidence for the translational validity of static late-phase SV2A PET quantification (39). From a clinical perspective, this supports the potential to reduce acquisition and analysis burden when dynamic imaging is not feasible, thereby increasing accessibility of SV2A PET. The added diagnostic value of dynamic acquisition, however, lies in its ability to extract early perfusion-like information, which cannot be obtained from static late-phase scans. This dual utility may position dynamic SV2A PET as a promising complementary imaging approach in presurgical epilepsy work-up.

Importantly, we systematically evaluated the impact of gaussian smoothing on lesion detectability and found that 8-mm FWHM provides an optimal trade-off between noise reduction and preservation of lesion volume. Such optimization has rarely been addressed in previous SV2A PET studies and may facilitate standardized image processing across sites and tracers.

Another important methodologic consideration concerns reference region selection. In addition to SUV-based analyses, we primarily used the cerebellum for early-phase scaling and the centrum semiovale for late-phase SUVR computation, in line with recent recommendations (15). This approach was informed by our own data, which demonstrated that cerebellar scaling yielded strong agreement with [^18^F]FDG uptake during the early postinjection period but declined thereafter. Additionally, 0–10 min [^18^F]UCB-H results were validated against alternative reference regions (whole brain and pons), yielding consistent findings across normalization strategies. During the late phase, tracer uptake primarily reflects specific SV2A binding in gray matter, whereas white matter showed predominantly nonspecific and stable signal, even after SV2A blockade, thereby supporting the use of the centrum semiovale as a reference region for late-phase SUVR computation (40,41). However, whereas the centrum semiovale is widely used because of its low synaptic density, its use should be cautiously interpreted in pathologies involving white matter and warrants further evaluation in larger cohorts of patients under SV2A-targeting medication.

Compared with other SV2A tracers such as [^11^C]UCB-J or [^18^F]SynVesT-2, [^18^F]UCB-H has been reported to exhibit lower affinity and slower kinetics (15). Although the present study was not designed as a formal pharmacologic blocking or occupancy experiment, our results nevertheless provide supportive evidence for in vivo SV2A target engagement, as indicated by pronounced reductions in [^18^F]UCB-H binding under SV2A-targeting treatment, consistent with observations from studies using alternative tracers (22). Interestingly, despite discontinuation of SV2A-binding medication in some patients before the PET scan, the extent of SV2A signal reduction in our human cohort was comparable to the approximately 40%–50% receptor occupancy previously reported in nonhuman primates and rodents (16,42) and even exceeded these values, with peak signal blocking of up to 75% in cortical subregions. These observations should be interpreted in the context of the observational, cross-sectional medication design with heterogeneous doses, treatment durations, and washout intervals and are not intended to provide definite quantitative estimates of SV2A occupancy. Nevertheless, the consistent and regionally specific signal reductions observed under SV2A-targeting medication underscore the high in vivo specificity of [^18^F]UCB-H for SV2A in humans.

Moreover, the quantitative results of our human SV2A PET examinations align well with previous findings using [^18^F]UCB-H in Alzheimer disease, further supporting the tracer’s reliability across different clinical populations (43). Head-to-head comparisons of [^18^F]UCB-H with other fluorinated SV2A tracers are currently unavailable. Therefore, the choice of tracer may be guided by availability, regulatory approval, and site-specific infrastructure rather than clear biologic superiority. Our data contribute to this gap by demonstrating that [^18^F]UCB-H yields reproducible and interpretable results in a clinical epilepsy population, supporting its continued use and evaluation.

This study has limitations. First, although our cohort is relatively large for dual PET studies, subgroup analyses, particularly in patients under SV2A-blocking medication, remain limited in statistical power. Second, histopathologic validation of SV2A reductions was not available. Third, the generalizability of reference region approaches and kinetic findings should be tested in broader and more heterogeneous epilepsy populations. Finally, no patient underwent epilepsy surgery after SV2A PET imaging during the observation period. Therefore, surgical outcome and correspondence between resected tissue and PET findings could not be assessed in this cross-sectional study.

Future work should focus on head-to-head comparisons of fluorinated SV2A tracers and the integration of SV2A PET with other modalities such as ictal SPECT, MRI, magnetoencephalography, or intracranial electroencephalography. A direct comparison of 0–10 min [^18^F]UCB-H with established SPECT and PET perfusion tracers will be essential to formally establish the extent to which early SV2A PET reflects regional cerebral blood flow. Longitudinal studies including pre- and postoperative imaging may also clarify whether SV2A PET can serve as a candidate biomarker for postsurgical outcome, treatment response, epileptogenicity, and disease progression in patients with epilepsy.

## CONCLUSION

[^18^F]UCB-H PET enables the simultaneous assessment of synaptic density and perfusion-like characteristics in focal epilepsy. Early-phase imaging offers perfusion-like information with spatial correspondence to [^18^F]FDG PET, whereas late-phase imaging provides high-contrast maps of synaptic integrity. Optimized normalization and filtering approaches further enhance lesion detectability. These findings support SV2A PET with [^18^F]UCB-H as a promising adjunct to [^18^F]FDG in the evaluation of focal epilepsy.

## DISCLOSURE

Johannes Gnörich is funded by the Munich Clinician Scientist Program (MCSP). Matthias Brendel is a member of the Neuroimaging Committee of the EANM; has received speaker honoraria from Roche, GE HealthCare, Iba, and Life Molecular Imaging; has advised Life Molecular Imaging and GE HealthCare; and is currently on the advisory board of MIAC. Nicholas Fearns has received speaker or consulting honoraria from Life Molecular Imaging, MSD, GE HealthCare, Eisai, and Biogen. Rudolf Werner has received speaker honoraria from Novartis/AAA and PentixaPharm and reports advisory board work for Novartis/AAA and Bayer. Andreas Zwergal has received speaker honoraria from Dr. Willmar Schwabe GmbH, Pfizer, and AstraZeneca and research support from Dr. Willmar Schwabe GmbH. No other potential conflict of interest relevant to this article was reported.

## Notes

KEY POINTS **QUESTION:** Can dual-phase SV2A PET imaging with [^18^F]UCB-H serve as a diagnostic alternative or complement to [^18^F]FDG PET in patients with focal epilepsy? **PERTINENT FINDINGS:** This dual-tracer study assessed dynamic [^18^F]UCB-H PET in 29 patients with pharmacoresistant unilateral focal epilepsy. Early-phase images (0–10 min) reflected perfusion-like uptake patterns and correlated with [^18^F]FDG lesion volume, whereas late-phase images (30–60 min) revealed more spatially confined synaptic signal changes. Pronounced signal reductions in patients receiving SV2A-binding antiseizure medication provided supportive evidence for in vivo target engagement. **IMPLICATIONS FOR PATIENT CARE:** SV2A PET imaging with [^18^F]UCB-H may enhance the presurgical evaluation of focal epilepsy by combining perfusion-like imaging and synaptic density assessment in a single scan.
